# The coordinated regulation mechanism of rice plant architecture and its tolerance to stress

**DOI:** 10.3389/fpls.2022.1087378

**Published:** 2022-12-15

**Authors:** Huibo Zhao, Xiong Liu, Jiajia Wang, Qian Qian, Guangheng Zhang

**Affiliations:** ^1^ State Key Laboratory of Rice Biology, China National Rice Research Institute, Hangzhou, China; ^2^ National Nanfan Research Institute (Sanya), Chinese Academy of Agricultural Sciences, Sanya, China

**Keywords:** rice, plant architecture, stress tolerance, biological breeding, coordinated regulation of multi genes

## Abstract

Rice plant architecture and stress tolerance have historically been primary concerns for rice breeders. The “Green Revolution” and super-rice breeding practices have demonstrated that ideal plant architecture can effectively improve both stress tolerance and yield. The synergistic selection and breeding of rice varieties with ideal architecture and stress tolerance can increase and stabilize yield. While rice plant plant architecture and stress tolerance are separately regulated by complicated genetic networks, the molecular mechanisms underlying their relationships and synergism have not yet been explored. In this paper, we review the regulatory mechanism between plant architecture, stress tolerance, and biological defense at the different level to provide a theoretical basis for the genetic network of the synergistic regulation and improvement of multiple traits.

## 1 Introduction

Global climate extremes profoundly affect human social and economic behavior, especially agricultural production in fragile ecosystems. In recent years, China’s rice production and food security have been challenged by natural disasters such as cold damage, high temperatures, and drought stress. Therefore, improving the stress tolerance of rice is important for overcoming these issues and ensuring food security.

The concept of ideal crop architecture was first proposed by Donald in 1968 (Donald, 1968). Ideal plant architecture can improve the photosynthetic efficiency, biomass, and stress tolerance of plants (Donald, 1968; Ma et al., 2020). Rice production continued to increase through two Green Revolutions and has been accompanied by improvements in architecture and tolerance (Khush, 2001). Traditional breeding and modern molecular breeding practices have demonstrated that there is a close relationship between plant architecture and stress tolerance, which complement each other throughout reproductive process for rice and jointly affect rice yield (Guo et al., 2020). Therefore, breeders have focused on rice plant architecture and stress tolerance, it is an effective way to achieve high and stable yield and solve the problem of food security to carry out the cooperative breeding of ideal plant types and stress tolerance.

## 2 Molecular regulation of rice plant architecture and its interrelationship with stress-tolerance

**Figure 1:**
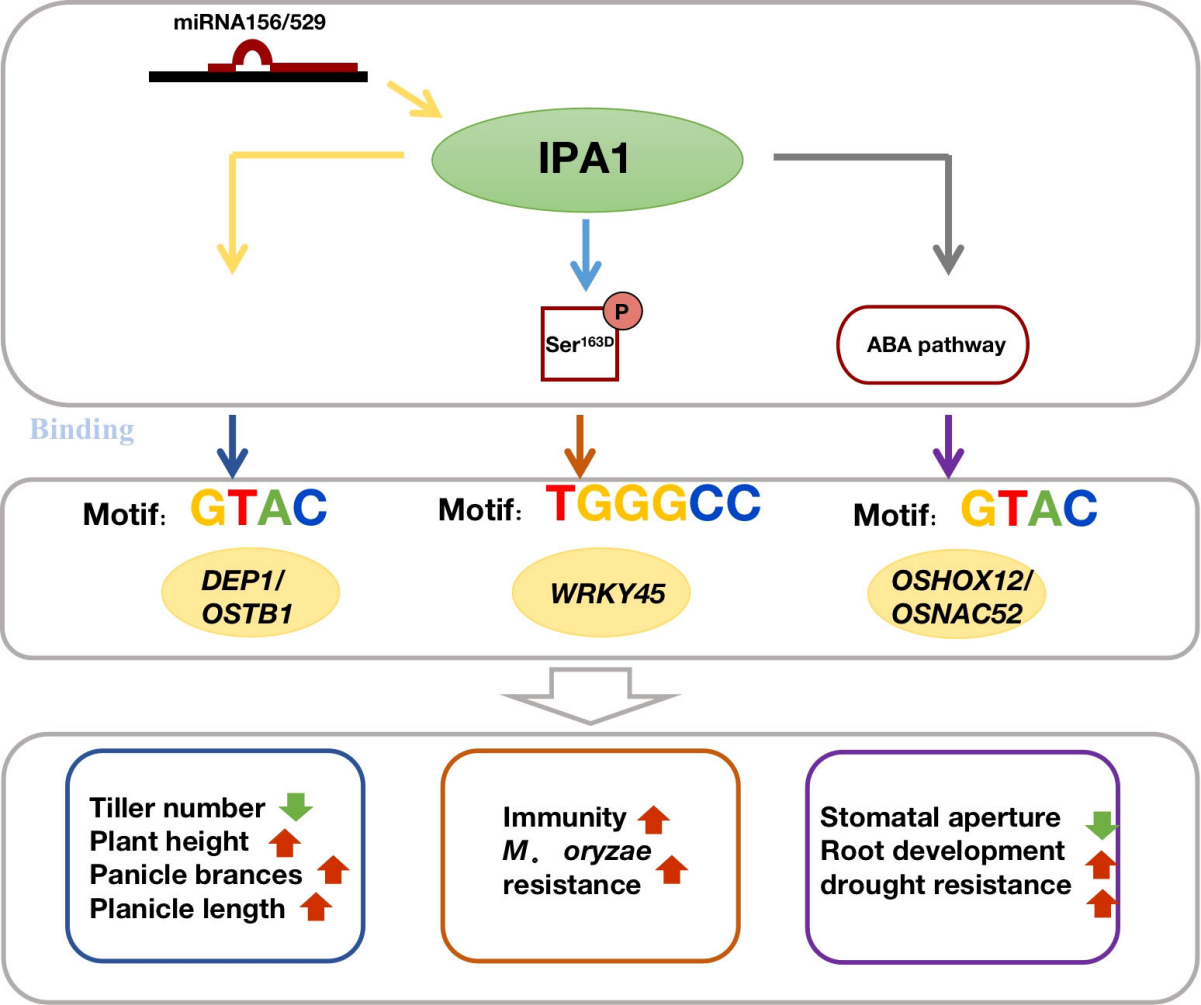
Regulatory relationship of *IPA1* on plant architecture and stress tolerance.

The acquisition of environmental awareness and tolerance in plants is a complicated process involving the coordinated action of many genes and multiple tolerance mechanisms, including a long period of domestication during evolution and a relatively short-term acclimation mechanism (Mittler et al., 2012). Traditional breeding practices have found that rice plant types with short stalks, thick stems, and upright spikes can strengthen the ability to resist lodging, while traits like small leaves, few tillers, and large spikes can effectively enhance drought resistance (Quarrie et al., 1997; Tu et al., 2022). The core technology of the first Green Revolution uses the semi-dwarf gene *Sd1* in rice breeding, which is the first variety to greatly increase production by reducing rice plant height and improving lodging resistance. It is also the first time to improve rice resistance by improving the plant type (Peng et al., 1999; Guo et al., 2020). Similarly, Liu et al. found that a height-, tiller-, and spike length-related regulatory gene *HTD2* (*D88*/*D14*) encodes an esterase that regulates cell growth and organ development through the strigolactone pathway. In rice, it inhibits the meristem and negatively regulate the tiller number, which helps regulate rice plant architecture (Liu et al., 2009; Wang et al., 2020). In 2010, Jiao et al. cloned a key gene *OsIPA1*, it encodes the Squamosa-like promoter-binding protein OsSPL14, which is regulated by *miR156* and binds to the important downstream rice plant type target genes *OsDEP1* and *OsTB1* and directly interacts with *OsSHI1* and *OsIPI*1 to co-regulate rice tiller, plant height, panicle type, and stem development. Therefore, increasing *OsIPA1* expression can reduce plant tiller, increase the grain number per panicle, and increase yield (Jiao et al., 2010; Lu et al., 2013; Wang et al., 2017; Wang and Wang, 2017; Duan et al., 2019). Meanwhile, under low-temperature stress, *OsTB1* and *OsMADS57* synergistically regulated the transcription of their target genes *OsWRKY94* and *D14*, shifting the morphological development of rice to cold adaptation and improving its cold resistance (Chen et al., 2018). In addition to regulating plant architecture development as a growth regulator, *IPA1* also positively regulates rice blast resistance by modulating amino acid phosphorylation at Ser^163^ to binds to the promoter of the pathogen defense gene *WRKY45*, maintaining the balance between growth and immunity (Wang et al., 2018). Additionally, *IPA1* improves drought resistance at the rice seedling stage by participating in ABA metabolism (Zhu et al., 2022). Functional analysis of *OsIPA1* confirms the possibility that the same gene could regulate both plant-type development and its abiotic stress tolerance characteristics (**Figure 1**).

Leaf morphology is an important factor affecting plant architecture. Several leaf-shape regulatory genes, such as *SLL1*, *PSL1*, and *SRL1*, were cloned by Zhang et al. using specific germplasm and mutants, which regulate the development of leaf polarity and play an important role in resisting adversity stress (Zhang et al., 2009; Xiang et al., 2012; Zhang et al., 2021). *SLL1*, a KANADI family transcription factor, affects leaf-rolling phenotypes by regulating the development of sclerenchyma cells on the abaxial surface of rice leaf and interacts with *OsSKIPa* to regulate drought tolerance in rice (Zhang et al., 2009; Hou et al., 2009). *PSL1*, the gene encoding *polygalacturonase*, changes the cell wall structure and water homeostasis *via* gene differential expression, thus regulating the drought resistance of rice. *SRL1*, which encodes a GPI-anchored protein, positively regulates drought tolerance in rice mainly through leaf curling caused by epigenetic inactivation and by controlling cell wall formation to further influence the epidermis and water homeostasis (Li et al., 2017). Moreover, as a dominant-negative gene, *REL1* primarily responds to drought stress in rice through the ABA pathway while regulating leaf rolling (Liang et al., 2018).

In addition to leaf morphology, plant height, spike shape, tiller, and root development are important morphological factors in regulating rice plant architecture and stress response. *OsSDG721* encodes a TRITHORAX-like protein that affects plant height and spike shape, and positively regulates salt tolerance in rice by regulating the methylation of *OsHKT1;5* (Jiang et al., 2018; Liu et al., 2021). *OsDRO1* is involved in the morphological development of rice roots. Higher expressed *OsDRO1* significantly promoted root morphology development by increasing the angle of rice roots, which improved water uptake capacity. Over-expression of *OsDRO1* in shallow-rooted germplasm could promote deeper root establishment, improve drought and lodging resistance, and enhance yield (Uga et al., 2013). *OsLIC1* regulates stress tolerance and traits such as leaf structure, plant height, tiller angle, and grain number by activating the BR signaling pathway in rice (Wang et al., 2008).

In recent years, in addition to the studies of *IPA1* on the genetic regulation network of rice plant type and resistance, several protein families such as Zinc-finger protein (ZFP), double-stranded RNA binding protein (DsRBP), and Heat shock protein (HSP) have been successfully cloned, which has clarified the synergistic regulatory functions of rice plant development, stress tolerance, and biological defense.

## 3 Zinc finger proteins synergistically regulation of plant architecture establishment and stress tolerance in rice

Zinc finger proteins are a class of nucleic acid-binding transcription factors that play important roles in plant growth and development, hormone regulation, stress response, and transcriptional regulation (Noman et al., 2019). *PROG1* encodes a C2H2-like zinc-finger protein and plays an important role in the domestication of stolon or slope growth to upright growth in rice. *PROG1* from both wild and cultivated rice has transcriptional activation activity, and the loss of *PROG1* function in cultivated rice not only improved rice plant architecture but also increased the spike number, significantly increasing yield (Jin et al., 2008; Tan et al., 2008). Huang et al. (2009) cloned the C2H2-type zinc finger protein coding gene *DST* from the broad-leaf salt, and drought-tolerant mutant *dst*, which also has transcriptional activation activity. By directly binding to the DBS sequence of the promoter of reactive oxygen species-related genes to regulate their expression and affect stomatal opening through ABA-independent pathway, thereby negatively regulating drought and salt tolerance in rice (Huang et al., 2009). *DSTreg1*, the semi-dominant allele of *DST*, competes to bind to the promoter region of *OsCKX2* in a dominant negative regulatory manner to reduce its expression, resulting in increased plant height, reduced tillering, and increased grain number per spike. At the same time, the function of *DSTreg1* is closely related to SAM activity (Li et al., 2013). *DCA1*, a transcriptional co-activator of *DST*, encodes a CHY-type zinc finger protein that can form a heterotetramer with *DST* to regulate stomatal opening and stress tolerance in plants by affecting the expression of hydrogen peroxide scavenging factors, such as Prx24 (Cui et al., 2015). Therefore, *DST* affects the development of leaf shape and spike shape and regulates abiotic stress response by regulating the expression of different downstream genes. *OsLIC1*, a gene encoding CCCH-type zinc finger protein, is a transcription factor with both transcriptional activation activity and RNA binding activity. *OsLIC1* regulates the development of traits such as leaf angle, plant height, tiller angle, and grain number per spike by activating BR signaling pathway in rice (Wang et al., 2008; Zhang et al., 2012). It also interacts with proteins such as OsBZR1, OsALDH2B1, and AOS2 to activate JA synthesis and signal transduction to regulate rice defense against abiotic and biotic stresses (Ke et al., 2020). *OsDRZ1* encodes another zinc finger protein involved in regulating rice plant type and drought stress. But unlike most of the reported zinc finger proteins, *OsDRZ1* has transcriptional repressive activity and could regulate stress response in rice by affecting the expression of drought-responsive genes such as *OsGLP1* (Yuan et al., 2018). Additionally, ZFP185, OsDHHC1, SNFL1, and other zinc-finger proteins were also involved in leaf morphology and stress response of rice plants through different pathways (Zhou et al., 2017; He et al., 2018).

## 4 RNA-binding proteins mediate the regulation of rice plant morphogenesis and stress tolerance

In addition to being the main components of ribosomes, RBPs are also involved in RNA processing, signal recognition, transcriptional activation, and developmental regulation. Most double-stranded RBPs (DsRBPs) contain two functional or catalytic domains and can participate in multiple regulatory pathways (Lu and Fedoroff, 2000), such as sRNA synthesis and regulation, plant architecture regulation, stress tolerance, and biological defense (Waterhouse et al., 2001; Raghuram et al., 2015). *AtHYL1*, the gene encoding DsRBP, mediates the post-transcriptional regulation of miRNAs and represses the translation of its target genes. *AtHYL1* participates in miRNA processing, synthesis, and accumulation and regulates plant responses to hormones such as ABA, IAA, and CK by interacting with *AtDCL1*, *AtSE*, and *AtHEN1*, which affects leaf morphogenesis and stress tolerance (Lu and Fedoroff, 2000; Yang et al., 2021). Currently, 12 double-stranded RNA-binding domain-containing proteins have been identified in rice, including 8 double-stranded RNA-binding proteins (DRBs) and 4 Dicer-like (DCL) proteins, which are mainly involved in establishing rice leaf polarity, sRNA biosynthesis, and biotic-stress-resistance regulation (Liu et al., 2005; Song et al., 2012; Raghuram et al., 2015). Of them, both *OsDCL1* and *OsDCL4* are involved in miRNA maturation and regulate leaf morphological development (Lu and Fedoroff, 2000), while *OsDCL1* negatively regulates the basal resistance of rice to *Pyricularia oryzae Cav (* Zhang et al., 2015 *).*. Analysis of *OsDRB2* in rice showed that defects of the *OsDRB2*-miR166-*OsHBs* pathway could play an important role in formation of the rolled leaf phenotype, Moreover, *OsDRB2* also regulated accumulation of *miR160*, *miR390*, and *miR396* and expressions of the genes involved in leaf polarity to affect leaf development (Yuan et al., 2022). As we know, Double-stranded RNA binding domain containing proteins play an integral role in all the small RNA pathways of the plants (Eamens et al., 2012). The expression levels of *OsDRB1-2*, *OsDRB1-3, OsDRB2* and *OsDRB3* genes were up-regulated in rice seedlings treated with abiotic treatments such as UV-B and drought and biological treatments. meanwhile, *DRB1* is a phosphorylation target of mitogen activated protein kinase MPK3 in both rice and *Arabidopsis*, and the transcripts of *OsMPKs* in rice were differentially regulated in abiotic and biological stresses, suggesting their stress-responsive functions as evident by the literature (Raghuram et al., 2015). This provided more new ideas for DRB to participate in rice stress response.

## 5 The functional mechanism of heat shock proteins in rice plant type development and stress tolerance

Nowadays, heat shock proteins are often involved in plant growth and development and various stress responses as molecular chaperones (Liu et al., 2021). More than 30 heat shock proteins have been reported in rice, but only *Nal11* and *OsHSF18* are involved in regulating rice plant type. *Nal11* encodes the small molecule heat shock protein HSP40. The disruption of the DNAJ domain in mutant *nal11* affects the mRNA splicing pattern and leads to premature termination of translation, which affects agronomic traits such as the tiller, leaf width, and panicle length in rice (Wu et al., 2016). Meanwhile, *Nal11* has been confirmed to have a negative regulatory effect on the drought resistance of rice seedlings in terms of morphology, physiology and biochemistry, and gene expression (Wang et al., 2020). Current studies have demonstrated that *OsHSF18* is involved in plant heat-resistance, cold-resistance, drought-resistance, and salt-resistance. RNA-Seq and ChIP-Seq screening revealed that *OsHSF18*-OX lines plants primarily respond to heat stress by participating in phytohormone signal transduction, ascorbic acid, and other metabolic pathways (Li, 2018). Meanwhile, that excessive transcription levels of *OsHSF18* negatively regulate plant height, tillering, seed setting rate, and 1000-grain weight (Qin, 2015). It is worth noting that the involvement of HSPs in the regulation of rice plant growth and development and stress tolerance is complex (since they are molecular chaperones), therefore, the regulatory mechanisms need to be further investigated.

## 6 Regulation of rice plant development and stress tolerance by hormones, miRNAs, and transcription factors

**Figure 2:**
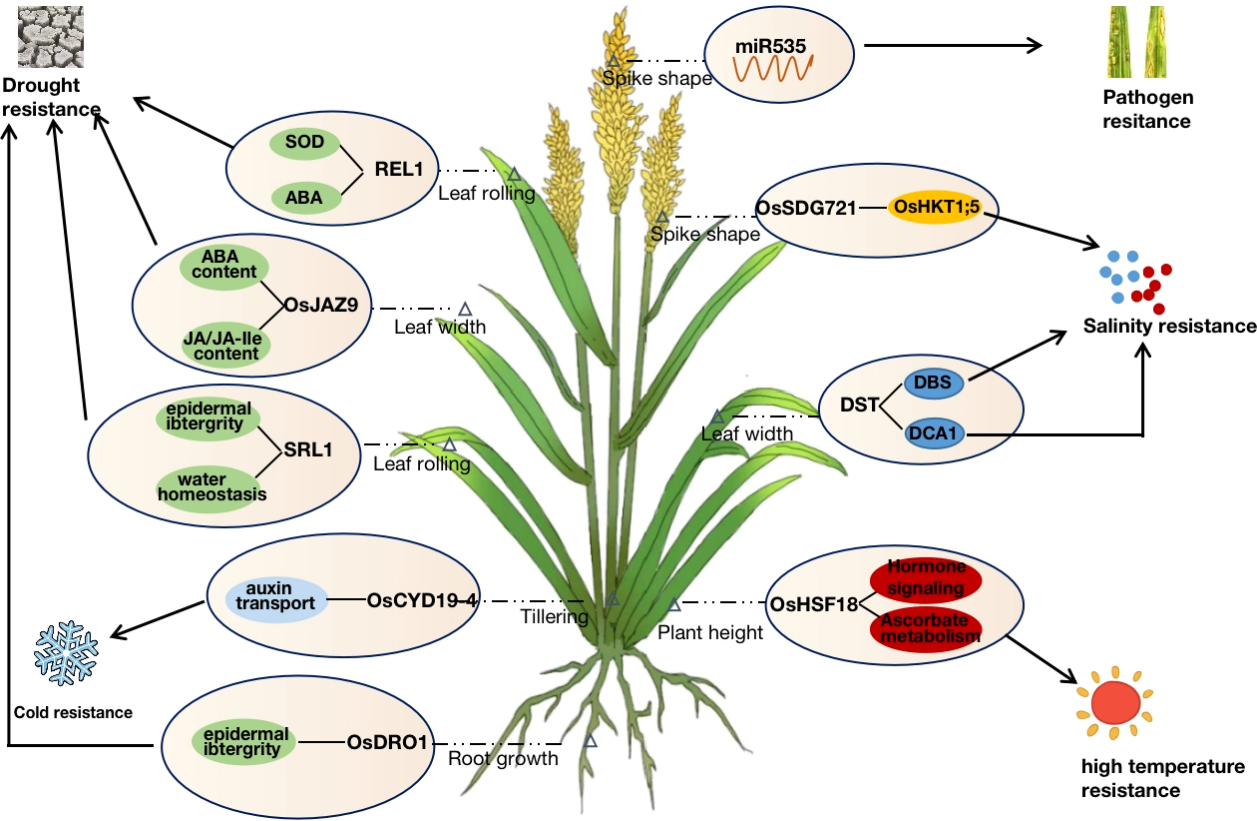
Multidimensional synergistic regulation of rice architecture and stress resistance.

Rice plant architecture and stress tolerance characteristics are simultaneously regulated by genetic, environmental, and protein levels. The genetic regulatory network is complicated, where most proteins cannot perform their functions alone but typically form complexes with different proteins or interact with upstream and downstream proteins (Oliver, 2000). At the same time, different hormones and miRNAs are also involved in various physiological activities in the cell or organism, synergistically regulating and maintaining the balance of plant morphological development and stress tolerance. JA and ABA are important hormones required for plant growth and development and stress response. *OsJAZ9* is a repressor of JA and can respond to drought stress by modulating JA signaling to alter potassium homeostasis or by decreasing leaf width and stomatal density to reduce leaf transpiration (Singh et al., 2021). The binding of *miRNA166* to its primary target, *OsHB4*, promotes the expression of genes related to cell wall formation. The *miRNA166* knockout lines and *OsHB4* overexpression lines showed leaf curling traits and reduced water conductivity due to a reduced diameter of stem xylem ducts, thus exhibiting higher drought tolerance (Zhang et al., 2018). Similarly, the F-box gene *MAIF1*, which is involved in the root growth of rice plants under miRNA regulation, is also induced by hormones such as ABA, JA, and CK to negatively regulate resistance to drought, salt, and low-temperature stresses (Yan et al., 2011). *OsCYP19-4* could be involved in rice plant development and cold stress adaptation by regulating auxin transport. The promoter of *OsCYP19-4* was activated when responding to cold stress. and the overexpression of *OsCYP19-4* caused a significant increase in the tiller number and spike number. Therefore, regulating *OsCYP19-4* expression could increase rice biomass or improve cold tolerance. *miR535* is involved in rice agronomic traits such as plant height and spike shape by regulating the expression of the *OsSPL* gene family (Sun et al., 2019) and negatively regulating immunity to rice blast (Li, 2017). In addition, the transcription factor family is particularly important in plant growth and development. *OsMYB91*, a transcription factor of R2R3-type MYB, participates in salt stress response *via* DNA demethylation and histone acetylation, while negatively regulating plant height in rice (Zhu et al., 2015). The MADS-box transcription factor *OsMADS25* regulates rice root development through the nitrate accumulation pathway and also enhances rice tolerance to low temperature and salt stress through an ABA-dependent signaling pathway (Yu et al., 2015; Xu, 2019; Yan et al., 2021) (**Figure 2**).

## 7 Discussion and outlook

Increasing rice production is important for global food security. However, the frequent occurrence of extreme weather events (e.g., high temperatures, low temperatures, droughts, and floods) caused by climate change poses a serious threat to rice production. The typical high and stable yield of rice is determined by its genotype and external environmental conditions. As a “smart” plant, rice constantly changes its plant type to adapt to different environmental conditions. For example, under high temperature conditions, rice plant reduces transpiration by promoting leaf curling, thereby enhancing resistance (Zhang et al., 2021); in cold temperature environments, rice plant enhances the stress tolerance by enlarging leaf width (Yang et al., 2013); and rice plant improves its salt tolerance or drought resistance by increasing the number of lateral roots, thereby improving the water absorption capacity of the root system (Seo et al., 2020); etc. In addition, plant architecture, which is determined by environmental conditions, is also involved in the regulation of the optimal planting density of rice. Proper leaf morphology and appropriate number of tillers contribute to a more efficient spatial arrangement and photosynthetic efficiency of rice plants, thus achieving both increased resistance to stress and yield of rice plants (Jun et al., 2006). The ability of rice plants to adapt to adversity through plant architecture improvement thus provides a new idea for future rice breeding. By combining conventional breeding methods to select for superior traits, and by using molecular techniques to explore the genes for superior traits and resolve the corresponding molecular regulatory mechanisms, new rice varieties with favorable stature and high tolerance to adversity can be selected more efficiently.

Rice has long been cultivated in China, making it rich in wild germplasm resources with a wide array of local germplasm varieties, salt-tolerant varieties, and deep rice varieties. These germplasm resources have accumulated abundant genetic resources during their natural evolution and artificial domestication. The mining, analysis, and utilization of these genetic resources are important for enhancing resistance and improving agronomic traits (Liu et al., 2018). With the discovery of *sd1* in “Dijiaowujian” realized the dwarfing profile of rice plants, which improved the lodging resistance of rice and catalyzed the first “Green Revolution” of rice production. It is easier to transfer good genes from wild rice because it is the ancestor of cultivated rice. Several genes and QTLs related to cold tolerance and drought resistance, such as *SRFP1* (Zhuang, 2016) and *OrbHLH00* (Li et al., 2010), have been mined and utilized in the wild rice variety “Dongxiang”. The DREB-like transcription factor, which has a typical AP2 structural domain, was successfully cloned from wild rice in “Chalin” and is a promising candidate for future cultivar selection to improve the resistance of cultivated rice to low-temperature stress (Liu, 2010). Because the acceleration of economic globalization and international trade liberalization, and current climate stresses have increased domestic and international demand for high-yield and high-quality rice, this requires using existing specific germplasm resources to explore and screen additional plants with highly resistant specific germplasm and favorable haplotypes.

Exploring more rational and advanced breeding approaches to develop new methods of rice breeding is also an essential mission for future rice breeding. Recent studies have found that variation in cis-regulatory regions (CRRs) can overcome pleiotropy among quantitative traits and provide a new source of targets for breeding beneficial traits, while the study of agronomic gene CRR systems will reveal more critical transcriptional regulatory networks, thus providing key information to guide the creation of novel elite alleles in plant breeding (Song et al., 2022). In 2021, Li Jiayang’s team achieved the domestication of heterotetraploid wild rice from scratch by using techniques such as multiple recombinant technology and gene-editing, providing a new breeding concept for a new rice with high yield and good environmental adaptation. This is also a new way of breeding practice for the synergistic improvement of rice plant development and stress tolerance (Yu et al., 2021).

Rice plant development and stress tolerance are complex agronomic traits controlled by multiple genes. To improve the synergistic regulatory network between plant development and stress tolerance, more genes must be identified. So far, plant breeding has made the leap from manual selection breeding, hybrid breeding to molecular breeding. In future r,esearch on rice production, it would be necessary to investigate the genetic network and signaling regulatory mechanism of rice plant development and stress response and apply them to breeding practice in a short period of time by combining various research methods. This includes QTL localization or Genome-Wide Association Studies (GWAS) to explore genes for superior agronomic traits and resistance genes, and multi-omics analyses such as transcriptomics, proteomics, metabolomics and epigenomics to investigate the regulatory network of plant development and resistance from a comprehensive perspective to achieve gene prediction and accurate breeding (Baldoni, 2022). To enable the utilization of genes for breeding, modern molecular methods such as CRISPR/Cas genome editing, marker-assisted selection (MAS), marker-assisted genealogical selection (MAPS), marker-assisted recurrent selection (MARS) and marker-assisted backcrossing (MABC) could be used to create, screen and identify desirable plant architecture and resistant germplasm resources (Oladosu et al., 2019). In addition, with the theory that extended light practices can shorten the plant growth cycle, speed breeding (SB) became the focus of attention (Samantara et al., 2022). By combining SB with conventional breeding or molecular breeding methods, SB can enhance the accuracy of plant phenotypic analysis while greatly shortening breeding time, which lays a good foundation for accelerating the synergistic improvement of rice architecture and resistance, and developing new high-yielding, high-quality, multi-resistant and environmentally friendly rice varieties.

## Author contributions

HZ and XL are the main writers of the article, JW participates in the production of the article pictures, and QQ and GZ participate in the correction of the article and provide fund project support. All authors contributed to the article and approved the submitted version.

## References

[B1] Baldoni E. (2022). Improving drought tolerance: Can comparative transcriptomics support strategic rice breeding? Plant Stress 3, 100058. doi: 10.1016/j.stress.2022.100058

[B2] Chen L. Zhao Y. Xu S. J. Zhang Z. Y. Xu Y. Y. Zhang J. Y. . (2018). OsMADS57 together with OsTB1 coordinates transcription of its target OsWRKY94 and D14 to switch its organogenesis to defense for cold adaptation in rice. New Phytol. 218 (1), 219–231. doi: 10.1111/nph.14977 29364524 PMC5873253

[B3] Cui L. G. Shan J. X. Shi M. Gao J. P. Lin H. X. . (2015). DCA1 acts as a transcriptional co-activator of DST and contributes to drought and salt tolerance in rice. PloS Genet. 11 (10), e1005617. doi: 10.1371/journal.pgen.1005617 26496194 PMC4619773

[B4] Donald C. M. (1968). The breeding of crop ideotypes. Euphytica 17 (3), 385–403. doi: 10.1007/BF00056241

[B5] Duan E. C. Wang Y. H. Li X. H. Lin Q. B. Zhang T. Wang Y. P. . (2019). OsSHI1 regulates plant architecture through modulating the transcriptional activity of IPA1 in rice. Plant Cell 31 (5), 1026–1042. doi: 10.1105/tpc.19.00023 30914468 PMC6533028

[B6] Eamens A. L. Wook Kim K. Waterhouse P. M. (2012). DRB2, DRB3 and DRB5 function in a non-canonical microRNA pathway in arabidopsis thaliana. Plant Signaling Behav. 7 (10), 1224–1229. doi: 10.4161/psb.21518 PMC3493401 22902697

[B7] Guo W. Chen L. Herrera-Estrella L. Cao D. Tran L. S. P. . (2020). Altering plant architecture to improve performance and resistance. Trends Plant Sci. 25 (11), 1154–1170. doi: 10.1016/j.tplants.2020.05.009 32595089

[B8] He P. Wang X. Zhang X. Jiang Y. D. Tian W. J. Zhang X. Q. . (2018). Short and narrow flag leaf1, a GATA zinc finger domain-containing protein, regulates flag leaf size in rice (Oryza sativa l.). BMC Plant Biol. 18 (1), 1–11. doi: 10.1186/s12870-018-1452-9 30413183 PMC6230254

[B9] Hou X. Xie K. Yao J. L. Qi Z. Y. Xiong L. Z. . (2009). A homolog of human ski-interacting protein in rice positively regulates cell viability and stress tolerance. Proc. Natl. Acad. Sci. 106 (15), 6410–6415. doi: 10.1073/pnas.0901940106 19339499 PMC2669339

[B10] Huang X. Y. Chao D. Y. Gao J. P. Zhu M. Z. Shi M. Lin H. X. . (2009). A previously unknown zinc finger protein, DST, regulates drought and salt tolerance in rice *via* stomatal aperture control. Genes Dev. 23 (15), 1805–1817. doi: 10.1101/gad.1812409 19651988 PMC2720257

[B11] Jiang P. Wang S. Ikram A. U. Xu Z. T. Jiang H. Y. Cheng B. J. . (2018). SDG721 and SDG705 are required for rice growth. J. Integr. Plant Biol. 60 (7), 530–535. doi: 10.1111/jipb.12644 29473711

[B12] Jiao Y. Wang Y. Xue D. W. Wang J. Yan M. X. Liu G. F. . (2010). Regulation of OsSPL14 by OsmiR156 defines ideal plant architecture in rice. Nat. Genet. 42 (6), 541–544. doi: 10.1038/ng.591 20495565

[B13] Jin J. Huang W. Gao J. P. Yang J. Shi M. Zhu M. Z. . (2008). Genetic control of rice plant architecture under domestication. Nat. Genet. 40 (11), 1365–1369. doi: 10.1038/ng.247 18820696

[B14] Jun M. A. Ming D. F. Yang S. M. Yang S. M. Zhu Q. S. . (2006). Characteristics of rice plant with heavy panicle. Agric. Sci. China 5 (12), 911–918. doi: 10.1016/S1671-2927(07)60004-2

[B15] Ke Y. Yuan M. Liu H. Hui S. Qin X. Chen J. . (2020). The versatile functions of OsALDH2B1 provide a genic basis for growth–defense trade-offs in rice. Proc. Natl. Acad. Sci. 117 (7), 3867–3873. doi: 10.1073/pnas.1918994117 32024752 PMC7035479

[B16] Khush G. S. (2001). Green revolution: the way forward. Nat. Rev. Genet. 2 (10), 815–822. doi: 10.1038/35093585 11584298

[B17] Li J. L. (2017). Preliminary study on the regulation of blast resistance and agronomic traits by 5 miRNAs such as osa-miR535 and osa-miR160a (Sichuan: Sichuan Agricultural University).

[B18] Li L. (2018). Functional mechanism of rice heat shock transcription factor OsHsf18 in regulating plant resistance to biotic and abiotic stress (Doctoral dissertation, Changsha: Hunan Agricultural University).

[B19] Liang J. Guo S. Sun B. Liu Q. Chen X. H. Peng H. F. . (2018). Constitutive expression of REL1 confers the rice response to drought stress and abscisic acid. Rice 11 (1), 1–11. doi: 10.1186/s12284-018-0251-0 30361842 PMC6202306

[B20] Li F. Guo S. Zhao Y. Chen D. Z. Chong K. Xu Y. Y. . (2010). Overexpression of a homopeptide repeat-containing bHLH protein gene (OrbHLH001) from dongxiang wild rice confers freezing and salt tolerance in transgenic arabidopsis. Plant Cell Rep. 29 (9), 977–986. doi: 10.1007/s00299-010-0883-z 20559833

[B21] Liu J. Y. (2010). Cloning of transcription factors related to freeze tolerance and study on the mechanism of freeze tolerance in chaling wild rice (Doctoral dissertation, Changsha: Hunan Agricultural University).

[B22] Liu G. F. Chen M. J. Li M. Lu H. Ge Y. Y Q. . (2018). Advances in rice breeding innovation. J. Plant Genet. Resour. 19 (3), 14. doi: 10.13430/j.cnki.jpgr.2018.03.006

[B23] Liu Y. Chen X. Xue S. Quan T. Y. Cui D. Han L. Z. . (2021). SET DOMAIN GROUP 721 protein functions in saline–alkaline stress tolerance in the model rice variety kitaake. Plant Biotechnol. J. 19 (12), 2576. doi: 10.1111/pbi.13683 34416090 PMC8633509

[B24] Liu B. Li P. C. Li X. Liu C. Y. Cao S. Y. Chu C. C. . (2005). Loss of function of OsDCL1 affects microRNA accumulation and causes developmental defects in rice. Plant Physiol. 139 (1), 296–305. doi: 10.1104/pp.105.063420 16126864 PMC1203379

[B25] Liu W. Wu C. Fu Y. Hu G. C. Si H. M. Zhu L. . (2009). Identification and characterization of HTD2: a novel gene negatively regulating tiller bud outgrowth in rice. Planta 230 (4), 649–658. doi: 10.1007/s00425-009-0975-6 19579033

[B26] Liu X. Y. Yang J. Liu J. Wang B. Dai L. Y. Li W. . (2021). Research progress of heat shock transcription factors in rice. Biotechnol. Bull. 37 (09), 226–233. doi: 10.13560/j.cnki.biotech.bull.1985.2020-1460

[B27] Li W. Q. Zhang M. J. Gan P. F. Qiao L. Yang S. Q. Miao H. . (2017). CLD 1/SRL 1 modulates leaf rolling by affecting cell wall formation, epidermis integrity and water homeostasis in rice. Plant J. 92 (5), 904–923. doi: 10.1111/tpj.13728 28960566

[B28] Li S. Y. Zhao B. R. Yuan D. Y. Duan M. Qian Q. Tang L. . (2013). Rice zinc finger protein DST enhances grain production through controlling Gn1a/OsCKX2 expression. Proc. Natl. Acad. Sci. 110 (8), 3167–3172. doi: 10.1073/pnas.1300359110 23382237 PMC3581943

[B29] Lu C. Fedoroff N. (2000). A mutation in the arabidopsis HYL1 gene encoding a dsRNA binding protein affects responses to abscisic acid, auxin, and cytokinin. Plant Cell 12 (12), 2351–2365. doi: 10.1105/tpc.12.12.2351 11148283 PMC102223

[B30] Lu Z. Yu H. Xiong G. Wang J. Jiao Y. Q. Liu G. F. . (2013). Genome-wide binding analysis of the transcription activator ideal plant architecture1 reveals a complex network regulating rice plant architecture. Plant Cell 25 (10), 3743–3759. doi: 10.1105/tpc.113.113639 24170127 PMC3877814

[B31] Ma M. G. Wen L. Kang X. M. Duan H. Y. . (2020). Research progress on improvement of ideal plant type of rice. Chin. Agric. Sci. Bull. 36 (29), 6.

[B32] Mittler R. Finka A. Goloubinoff P. (2012). How do plants feel the heat? Trends Biochem. Sci. 37 (3), 118–125. doi: 10.1016/j.tibs.2011.11.007 22236506

[B33] Noman A. Aqeel M. Khalid N. Islam W. Sanaullah T. Anwar M. . (2019). Zinc finger protein transcription factors: Integrated line of action for plant antimicrobial activity. Microbial Pathogenesis 132, 141–149. doi: 10.1016/j.micpath.2019.04.042 31051192

[B34] Oladosu Y. Rafii M. Y. Samuel C. Fatai A. Magaji U. Kareem I. . (2019). Drought resistance in rice from conventional to molecular breeding: a review. Int. J. Mol. Sci. 20 (14), 3519. doi: 10.3390/ijms20143519 31323764 PMC6678081

[B35] Oliver S. (2000). Guilt-by-association goes global. Nature 403 (6770), 601–602. doi: 10.1038/35001165 10688178

[B36] Peng J. Richards D. E. Hartley N. M. Murphy G. P. Devos K. M. Flintham J. E. . (1999). ‘Green revolution’ genes encode mutant gibberellin response modulators. Nature 400 (6741), 256–261. doi: 10.1038/22307 10421366

[B37] Qin H. Y. (2015). Function of rice heat shock transcription factor OsHSF18 in regulating stress resistance of rice (Doctoral dissertation, Changsha: Hunan Agricultural University).

[B38] Quarrie S. A. Laurie D. A. Zhu J. Lebreton C. Semikhodskii A. Steed A. . (1997). QTL analysis to study the association between leaf size and abscisic acid accumulation in droughted rice leaves and comparisons across cereals. Oryza: From Molecule to Plant, 155–165. doi: 10.1007/978-94-011-5794-0_15 9291969

[B39] Raghuram B. Sheikh A. H. Rustagi Y. Sinha A. K. . (2015). Micro RNA biogenesis factor DRB1 is a phosphorylation target of mitogen activated protein kinase MPK3 in both rice and arabidopsis. FEBS J. 282 (3), 521–536. doi: 10.1111/febs.13159 25417716

[B40] Samantara K. Bohra A. Mohapatra S. R. Prihatini R. Asibe F. Singh L. . (2022). Breeding more crops in less time: A perspective on speed breeding. Biology 11 (2), 275. doi: 10.3390/biology11020275 35205141 PMC8869642

[B41] Seo D. H. Seomun S. Choi Y. D. Jang G. . (2020). Root development and stress tolerance in rice: the key to improving stress tolerance without yield penalties. Int. J. Mol. Sci. 21 (5), 1807. doi: 10.3390/ijms21051807 32155710 PMC7084713

[B42] Singh A. P. Mani B. Giri J. (2021). OsJAZ9 is involved in water-deficit stress tolerance by regulating leaf width and stomatal density in rice. Plant Physiol. Biochem. 162, 161–170. doi: 10.1016/j.plaphy.2021.02.042 33684775

[B43] Song X. Li P. Zhai J. X. Zhou M. Ma L. J. Liu B. . (2012). Roles of DCL4 and DCL3b in rice phased small RNA biogenesis. Plant J. 69 (3), 462–474. doi: 10.1111/j.1365-313X.2011.04805.x 21973320

[B44] Song X. Meng X. Guo H. Cheng Q. Jing Y. H. Chen M. J. . (2022). Targeting a gene regulatory element enhances rice grain yield by decoupling panicle number and size. Nat. Biotechnol. 40, 1403–1411. doi: 10.1038/s41587-022-01281-7 35449414

[B45] Sun M. Shen Y. Li H. Y. Yang J. K. Cai X. X. Zheng G. P. . (2019). The multiple roles of OsmiR535 in modulating plant height, panicle branching and grain shape. Plant Sci. 283, 60–69. doi: 10.1016/j.plantsci.2019.02.002 31128716

[B46] Tan L. B. Li X. R. Liu F. X. Sun X. F. Li C. G. Zhu Z. F. . (2008). Control of a key transition from prostrate to erect growth in rice domestication. Nat. Genet. 40 (11), 1360–1364. doi: 10.1038/ng.197 18820699

[B47] Tu B. Tao Z. Wang S. Zhou L. Zheng L. Zhang C. . (2022). Loss of Gn1a/OsCKX2 confers heavy-panicle rice with excellent lodging resistance. J. Integr. Plant Biol. 64 (1), 23–38. doi: 10.1111/jipb.13185 34783157

[B48] Uga Y. Sugimoto K. Ogawa S. Rane J. Ishitani M. Hara N. . (2013). Control of root system architecture by DEEPER ROOTING 1 increases rice yield under drought conditions. Nat. Genet. 45 (9), 1097–1102. doi: 10.1038/ng.2725 23913002

[B49] Wang F. Han T. Song Q. Ye W. X. Song X. G. Chu J. F. . (2020). The rice circadian clock regulates tiller growth and panicle development through strigolactone signaling and sugar sensing. Plant Cell 32 (10), 3124–3138. doi: 10.1105/tpc.20.00289 32796126 PMC7534462

[B50] Wang B. Wang H. (2017). IPA1: a new “green revolution” gene? Mol. Plant 10 (6), 779–781. doi: 10.1016/j.molp.2017.04.011 28478096

[B51] Wang L. Xu Y. Zhang C. Ma Q. Joo S. H. Kim S. K. . (2008). OsLIC, a novel CCCH-type zinc finger protein with transcription activation, mediates rice architecture *via* brassinosteroids signaling. PloS One 3 (10), e3521. doi: 10.1371/journal.pone.0003521 18953406 PMC2567845

[B52] Wang J. Yu H. Xiong G. Lu Z. F. Jiao Y. Q. Meng X. B . (2017). Tissue-specific ubiquitination by IPA1 INTERACTING PROTEIN1 modulates IPA1 protein levels to regulate plant architecture in rice. Plant Cell 29 (4), 697–707. doi: 10.1105/tpc.16.00879 28298520 PMC5435429

[B53] Wang C. L. Zhang L. Luo L. X. Wang H. Guo T. Liu Y. Z. . (2020). Response analysis of rice NAL11 gene to abiotic stress at seedling stage. Acta Agriculturae Boreali-Sinica 35 (4), 9. doi: CNKI:SUN:HBNB.0.2020-04-017

[B54] Wang J. Zhou L. Shi H. Chern M. Yu H. Yi H. . (2018). A single transcription factor promotes both yield and immunity in rice. Science 361 (6406), 1026–1028. doi: 10.1126/science.aat7675 30190406

[B55] Waterhouse P. M. Wang M. B. Lough T. (2001). Gene silencing as an adaptive defence against viruses. Nature 411 (6839), 834–842. doi: 10.1038/35081168 11459066

[B56] Wu Y. Luo L. Chen L. Tao X. X. Huang M. Wang H. . (2016). Chromosome mapping, molecular cloning and expression analysis of a novel gene response for leaf width in rice. Biochem. Biophys. Res. Commun. 480 (3), 394–401. doi: 10.1016/j.bbrc.2016.10.061 27771249

[B57] Xiang J. J. Zhang G. H. Qian Q. Xue H. W. . (2012). Semi-rolled leaf1 encodes a putative glycosylphosphatidylinositol-anchored protein and modulates rice leaf rolling by regulating the formation of bulliform cells. Plant Physiol. 159 (4), 1488–1500. doi: 10.1104/pp.112.199968 22715111 PMC3425193

[B58] Xu N. (2019). Function of MADS-box transcription factor OsMADS25 in rice root development and response to high salt stress (Doctoral dissertation, Chongqing: Chongqing University).

[B59] Yan Y. S. Chen X. Y. Yang K. Sun Z. X. Fu Y. P. Zhang Y. M. . (2011). Overexpression of an f-box protein gene reduces abiotic stress tolerance and promotes root growth in rice. Mol. Plant 4 (1), 190–197. doi: 10.1093/mp/ssq066 21059694

[B60] Yang X. Dong W. Ren W. Zhao Q. X. Wu F. J. He Y. K. . (2021). Cytoplasmic HYL1 modulates miRNA-mediated translational repression. Plant Cell 33 (6), 1980–1996. doi: 10.1093/plcell/koab090 33764452 PMC8290291

[B61] Yang C. Li D. Mao D. Liu X. Ji C. G. Li X. B. . (2013). Overexpression of micro RNA 319 impacts leaf morphogenesis and leads to enhanced cold tolerance in rice (O ryza sativa l). Plant Cell Environ. 36 (12), 2207–2218. doi: 10.1111/pce.12130 23651319

[B62] Yan L. Y. Yuan D. Y. Duan M. J. Zhang H. J. Zheng Y. Q. Cong Y. Q. . (2021). Transcription factor OsMADS25 enhances low temperature tolerance in rice. Genetics 043 (011), 1078–1087. 10.16288/j.yczz.21-217 34815210

[B63] Yuan X. Huang P. Wang R. Q. Li H. Y. Lv X. Q. Duan M. . (2018). A zinc finger transcriptional repressor confers pleiotropic effects on rice growth and drought tolerance by down-regulating stress-responsive genes. Plant Cell Physiol. 59 (10), 2129–2142. doi: 10.1093/pcp/pcy133 30020522

[B64] Yuan Z. Pan J. Chen C. Tang Y. L. Zhang H. S. Guo J. . (2022). DRB2 modulates leaf rolling by regulating accumulation of MicroRNAs related to leaf development in rice. Int. J. Mol. Sci. 23 (19), 11147. doi: 10.3390/ijms231911147 36232465 PMC9570175

[B65] Yu H. Lin T. Meng X. Du H. L. Zhang J. K. Liu G. F. . (2021). A route to *de novo* domestication of wild allotetraploid rice. Cell 184 (5), 1156–1170.e14. doi: 10.1016/j.cell.2021.01.013 33539781

[B66] Yu C. Liu Y. Zhang A. D. Su S. Yan A. Huang L. L. . (2015). MADS-box transcription factor OsMADS25 regulates root development through affection of nitrate accumulation in rice. PloS One 10 (8), e0135196. doi: 10.1371/journal.pone.0135196 26258667 PMC4530940

[B67] Zhang G. Hou X. Wang L. Xu J. Chen. J. Fu X. . (2021). PHOTO-SENSITIVE LEAF ROLLING 1 encodes a polygalacturonase that modifies cell wall structure and drought tolerance in rice. New Phytol. 229 (2), 890–901. doi: 10.1111/nph.16899 32858770

[B68] Zhang D. Liu M. Tang M. Dong B. Wu D. X. Zhang Z. G. . (2015). Repression of microRNA biogenesis by silencing of OsDCL1 activates the basal resistance to magnaporthe oryzae in rice. Plant Sci. 237, 24–32. doi: 10.1016/j.plantsci.2015.05.002 26089149

[B69] Zhang C. Xu Y. Guo S. Zhu J. Huan Q. Liu H. . (2012). Dynamics of brassinosteroid response modulated by negative regulator LIC in rice. PloS Genet. 8 (4), e1002686. doi: 10.1371/journal.pgen.1002686 22570626 PMC3343102

[B70] Zhang G. H. Xu Q. Zhu X. D. Qian Q. Xue H. W. . (2009). SHALLOT-LIKE1 is a KANADI transcription factor that modulates rice leaf rolling by regulating leaf abaxial cell development. Plant Cell 21 (3), 719–735. doi: 10.1105/tpc.108.061457 19304938 PMC2671703

[B71] Zhang J. Zhang H. Srivastava A. K. Pan Y. J. Bai J. J. Fang J. J. . (2018). Knockdown of rice microRNA166 confers drought resistance by causing leaf rolling and altering stem xylem development. Plant Physiol. 176 (3), 2082–2094. doi: 10.1104/pp.17.01432 29367235 PMC5841683

[B72] Zhou B. Lin J. Z. Peng D. Yang Y. Z. Liu X. M . (2017). Plant architecture and grain yield are regulated by the novel DHHC-type zinc finger protein genes in rice (Oryza sativa l.). Plant Sci. 254, 12–21. doi: 10.1016/j.plantsci.2016.08.015 27964781

[B73] Zhuang Z. (2016). Identification and analysis of SRFP1 gene in dongxiang wild rice (Doctoral dissertation, Jiangxi: Jiangxi Agricultural University).

[B74] Zhu N. Cheng S. Liu X. Du H. Dai M. Q. Zhou D. X. . (2015). The R2R3-type MYB gene OsMYB91 has a function in coordinating plant growth and salt stress tolerance in rice. Plant Sci. 236, 146–156. doi: 10.1016/j.plantsci.2015.03.023 26025528

[B75] Zhu M. He Y. Zhu M. Ahmad A. Xu S. He Z. J. . (2022). ipa1 improves rice drought tolerance at seedling stage mainly through activating abscisic acid pathway. Plant Cell Rep. 41 (1), 221–232. doi: 10.1007/s00299-021-02804-3 34694441

